# Schools as They Are and as They Should Be

**Published:** 1847-02

**Authors:** H. M. Bullitt

**Affiliations:** Professor of Physiology and Pathology in the Medical Department of St. Louis University


3. *Schools as they are and as they should be.* By H. M. Bullitt, M.D., Professor of Physiology and Pathology in the Medical Department of St. Louis University.

Medical education is a subject in which all classes of men should feel deeply interested. The demand for physicians is a public demand, and not a mere professional requirement. The rapid increase of population in the United States has created such a call for doctors, that each year there seems to have arisen further necessity for the extension of the facilities of acquiring medical instruction. This necessity has been recognized not alone by the profession, but as well by the public, at whose cost indeed many of the medical institutions have been established. Every principal city in the Union accordingly has its school, and some of them are provided with several; so that the supply of schools, at least, seems to be now equal to the demand for doctors. Now, whilst we are willing to admit that there has been, and will continue to be, an annually increasing demand for physicians, to supply the growing population of the new States, and that this demand creates a demand for new schools, we doubt much whether the schools that are springing up in all directions, are the description of institutions which are calculated to supply the country with such physicians as it is entitled to. As a general rule, these institutions are established by, or at the instance of physicians who have either been disappointed in practice, or who for some reason have never become practitioners; restless, disappointed men, whose sole object is lucre, and who regard medical teaching as a traffic, to be carried on as other kinds of trade, merely for money, and who look upon the schools as business firms, to be sustained by hard puffing, diligent electioneering, or *drumming*, and extensive advertising. With such men it is a matter of little moment whether the students who may be attracted by this canvassing and advertising, are educated or not. They regard the relation of student and teacher as precisely similar to that of buyer and seller, and make the rule "caveat emptor," the standard of their moral obligations. Institutions which are in the hands of such persons cannot of course be suitable establishments for the education of physicians. The students of medicine generally are young men whose experience in the affairs of life is necessarily limited, and whose ability to judge of the qualifications of teachers of medicine is still more limited. They start from their homes with the determination to enter as pupils of some school which has been recommended by their preceptors, but before they can reach their destination they are often compelled to pass through some one or more places where there are schools of medicine, which, like other business firms, have their *drummers* stationed about in all directions, by whom every youth who looks like a medical student is to be beset with soft words and winning solicitations. Thus inexperienced, students are continually diverted from their original detornations, and induced, in disregard of the advice of their preceptors, to matriculate and disgorge the price of their tuition into the coffers of an institution of which they have never heard, and are left, perhaps, to spend their winter under auspices so little favorable to improvement, that they return in the spring, with nothing gained but a bundle of *tickets*. Or it may be that they have left their homes with directions to become the pupils of a particular school in a certain city, which they may be fortunate enough to reach without being interrupted on their way. Here perhaps there is another school than that to which they have been consigned, and it often happens that they are scarcely set down at the hotel of their choice before they are approached by some *kind hearted, disinterested* personage, who volunteers his advice, which is to disregard that of their preceptors, and become the pupils of another school; which, in the estimation of the adviser is infinitely superior to that to which they may have been consigned. This *purports*, of course, to be the advice of a *wholly disinterested* man, who is presumed, from his residence on the spot, to be perfectly familiar with the comparative merits of the schools. The reader doubtless recognizes in this personage the "drummer" of the school whose interest he so insidiously advocates. Well, the unsuspecting students are induced at last to visit the school thus highly recommended by this unknown *friend*, and compare its teachers with those of the school which their *true friend*, their preceptor, has recommended; and thus they are seduced into the clutches of these *professional speculators.* The result may be easily foretold. If they be susceptible of flattery, as most young men are, the superlatively condescending attentions of these professional dignitaries will so charm and fascinate them, that they will find it almost impossible to resist the solicitations to matriculate, and take their "tickets," even before they have visited the school of their deliberate choice at all. No comparison, therefore, is made; but even were this attempted, students are not qualified to decide between good and profitable teaching and its more fascinating semblance. These are not mere fancy sketches, drawn for amusement, nor for sinister purposes, but veritable pictures, as true to facts as the most faithful Daguerreotype can be to nature.

Now in view of the dangers by which students are thus beset, two things become important; first, that private preceptors should regard it as one of the most imperative duties to their pupils to select a school for them, and guard them effectually against the danger of being seduced off from this school of their choice. The pupils should be made to understand that certain schools will use every means which avarice can suggest to obtain their money by securing them as pupils; and they should be especially instructed that such a course is indicative at once of want of professional pride, and of conscious inferiority. I hold that no honorable man can descend so low as to use such means, and no man of real merit as a teacher can do it; because true Science imparts to her faithful followers a dignity and elevation of character which would revolt at the idea of deception or injustice. An honorable man, such as the true lover of Science is always found to be, regards the money with which a pupil starts from his home as already the property of those to whose hands he has been consigned, and would look upon himself as little better than a swindler were he to become possessed of it by seducing the pupil from the course advised by the preceptor. Such interference with the rights of the private preceptor or with the interests of the school recommended by him, must elicit the scorn and contempt of all honorable men. A true lover of Science, conscious of his own merit, would spurn the thought of gathering a class by any such means. Let every physician, therefore, who undertakes the responsible office of private preceptor, make it his especial duty to select from among the various schools of the country the one which in his judgment may seem most worthy, and use his influence with his pupils to induce them to finish their course of study in such institution. In this way the schools will be judged by those whose finished education is presumed to qualify them for the task. The teachers must thus stand or fall by the judgment of their peers. And mere speculators in the business of medical teaching will less easily build up their schools by means of flattery and deception.

But it may be said that those who are likely to be the private preceptors of the majority of pupils can not, from their remote situations, in the villages and country places, have opportunities of forming just estimates of the abilities of teachers whom they have never heard lecture, and that they must therefore leave it with the pupil to decide amongst the schools according to the information they may get after leaving home, aided by their own observation. We must confess we do not see the force of this objection to the course we have recommended; for there are various means of estimating the merit of a school, within the reach of every physician. In the first place, every public teacher should be a contributor to the cause of the profession, through some one or more of the medical journals, and thus afford the profession some evidence of his merit. But the best method of estimating the desert of a school, is by the proficiency of its alumni. Most of the schools are yearly sending forth graduates to all parts of the country, and the attainments and success of these, as they are exhibited while they are yet fresh from the colleges, is perhaps the best criterion of the worthiness of their alma mater. But granting that the private preceptors are not in possession of the means of judging between the schools, it becomes necessary in that case that they should instruct their students as to what constitutes good teaching. The pupil should be made to understand that the mere manner and voice of the lecturer have less to do with the business of scientific teaching than young men are prone to suppose. A musical voice and stump orator manner will please, perhaps fascinate the mind of the inexperienced student; but these are far from being necessary, or even commendable in scientific lecturers. The style of the best and most esteemed teachers of science is entirely conversational, and necessarily so, because it is only when the speaker begins to theorize, or rant, or moralize that the manner and cadences of the stump orator or the forensic or pulpit orators ever can be advantageously employed. There are so many different styles of lecturing in the different medical schools of our Union, that it is admitted to be a difficult matter to determine the exact degree of faultiness of the bad; but we can apply to them the method of exclusion, and thus be able to determine when they are not good. This is to be accomplished by indicating the styles which are adopted by the best teachers and found to be best in practice. But as this will require some care and reflection, we shall postpone its consideration for the present.—*St. Louis Med. & Surg. Journal.*

